# Circadian Variation in the Response to Vaccination: A Systematic Review and Evidence Appraisal

**DOI:** 10.1177/07487304241232447

**Published:** 2024-03-08

**Authors:** Cathy A. Wyse, Laura M. Rudderham, Enya A. Nordon, Louise M. Ince, Andrew N. Coogan, Lorna M. Lopez

**Affiliations:** * Kathleen Lonsdale Institute for Human Health Research and Department of Biology, Maynooth University, Maynooth, Ireland; † Division of Pharmacology and Toxicology, College of Pharmacy, The University of Texas at Austin, Austin, Texas, USA,; ‡ Kathleen Lonsdale Institute for Human Health Research and Department of Psychology, Maynooth University, Maynooth, Ireland

**Keywords:** circadian, time of day, vaccine, adaptive immunity

## Abstract

Molecular timing mechanisms known as circadian clocks drive endogenous 24-h rhythmicity in most physiological functions, including innate and adaptive immunity. Consequently, the response to immune challenge such as vaccination might depend on the time of day of exposure. This study assessed whether the time of day of vaccination (TODV) is associated with the subsequent immune and clinical response by conducting a systematic review of previous studies. The Cochrane Library, PubMed, Google, Medline, and Embase were searched for studies that reported TODV and immune and clinical outcomes, yielding 3114 studies, 23 of which met the inclusion criteria. The global severe acute respiratory syndrome coronavirus 2 vaccination program facilitated investigation of TODV and almost half of the studies included reported data collected during the COVID-19 pandemic. There was considerable heterogeneity in the demography of participants and type of vaccine, and most studies were biased by failure to account for immune status prior to vaccination, self-selection of vaccination time, or confounding factors such as sleep, chronotype, and shiftwork. The optimum TODV was concluded to be afternoon (5 studies), morning (5 studies), morning and afternoon (1 study), midday (1 study), and morning or late afternoon (1 study), with the remaining 10 studies reporting no effect. Further research is required to understand the relationship between TODV and subsequent immune outcome and whether any clinical benefit outweighs the potential effect of this intervention on vaccine uptake.

In 1798, Edward Jenner reported that infection with cowpox conferred immunity to smallpox, an observation that yielded a prophylactic tool that would eliminate the disease from the world by 1980. Today, vaccination is a key component of primary health care and a human right that prevents around 4-5 million deaths per year as reported by the World Health Organization (WHO, 2023). However, at an individual level, the effectiveness of vaccination can be compromised by poor immunological responses in those most vulnerable to infection, including older adults, those who are immunocompromised, and people with obesity (Zimmermann and Curtis, 2019). Interventions that enhance vaccine effectiveness could help to improve clinical outcomes and to optimize the control and global elimination of infectious disease.

Circadian rhythms are daily oscillations in physiology that are driven by feedback loops in the transcription and translation of a panel of “clock” genes and other biochemical timing mechanisms that are present virtually in every human cell, including immune cells (Takahashi, 2017). The concept of circadian rhythmicity in immunity implies that there are times of day that immune defense and resilience to infection are heightened and survival is optimized. In support of this, daily windows of increased susceptibility to viral infection and to the lethal effects of sterile inflammatory challenge have been demonstrated in animal models (Edgar et al., 2016; Halberg et al., 1960; Sengupta et al., 2019).

Similarly, the immune response to vaccination has been shown in animal studies to be dependent on the time of day; mice vaccinated toward the end of their resting phase (day-time) showed increased T-cell activation and proliferation (Fortier et al., 2011; Ince et al., 2023; Nobis et al., 2019), migration of dendritic cells into the lymph nodes (Holtkamp et al., 2021), germinal center B-cells, and circulating antibodies (Ince et al., 2023) compared to those vaccinated in their active phase. However, there is conflicting evidence for the optimum time of day of vaccination (TODV) in mice, with some studies reporting increased antigen-specific lymphocyte proliferation (Silver et al., 2012), elevation of antigen-specific antibodies, germinal center B-cells, and follicular helper T cells (Suzuki et al., 2016) after vaccination in the active (night-time) phase. Most of these animal studies demonstrated persistence of the effects of TODV in constant conditions and abrogation or attenuation in clock-deficient animals, supporting direct regulation of the immune response to vaccination by the circadian clock.

There is accumulating evidence for comparable circadian variation in human immune function; circadian oscillation in clock gene expression has been demonstrated in human peripheral blood mononuclear cells (Boivin et al., 2003) and in CD4+ T cells (Bollinger et al., 2011) and functional immune rhythms were suggested by diurnal patterns in interleukin (IL)-2, IL-4, and interferon-γ production in ex vivo–stimulated human CD4+ T cells (Bollinger et al., 2011). There is considerable evidence for circadian regulation of the innate immune system, including circadian oscillation of clock gene expression in phagocytic cells (Nguyen et al., 2013; Timmons et al., 2020), and variation in recruitment of neutrophils to sites of inflammation (Gibbs et al., 2014).

Studies in UK Biobank reported population-level diurnal variation in white blood cells and inflammatory markers that were independent of demographic and lifestyle confounding factors (Wyse et al., 2021). Despite the convincing evidence of the importance of TODV in mouse models, there is much discrepancy between studies of the timing of vaccination in human medicine. In contrast to animal models, the assessment of the effect of TODV in humans is confounded by many lifestyle factors that show daily variation (e.g., work, stress, mealtimes, antigen exposure) that could mask an effect of endogenous circadian rhythms in immune function on response to vaccination. Furthermore, the time-of-day preference of an individual (chronotype) is associated with genetics (Jones et al., 2019), health, and age (Knutson and von Schantz, 2018) and could link the TODV to vaccination outcome independent of any underlying circadian rhythm in immune function. Assessment of an effect of TODV in humans must account for multiple confounding factors that affect human immune function such as age (Wu et al., 2022), sex (Zimmermann and Curtis, 2019), sleep (Lange et al., 2011), shift work (Ruiz et al., 2020), vaccination history (Tsang et al., 2014), and co-morbidity (Zimmermann and Curtis, 2019).

Time-dependent responses to vaccination might be caused by endogenous rhythms that serve to optimize immune function at specific times of day. Vaccination is an elective immune challenge that could theoretically be aligned with an optimal circadian phase to improve effectiveness, but this would also present a logistic obstacle to mass-vaccination and could undermine public confidence in vaccination at times of day proposed to be less favorable. Here we report a systematic review of studies that investigated human immune response to vaccination at different times of day and assess the evidence to support diurnal variation in the effectiveness of vaccination.

## Methods

### Literature Searches

A protocol for this review was registered in the International Prospective Register of Systematic Reviews (CRD42023401086) and this review is reported according to the Preferred Reporting Items for Systematic Reviews and Meta-Analyses statement (Matryba et al., 2022). We searched the following 4 databases: PubMed, Embase, Cochrane Library, and Medline with no restriction on the time of publication. The search was limited to the English language and included preprint publications and theses. The search terms and MESH headings for all databases are available in the Supplementary Material. The reference lists of relevant reviews and of all included studies were hand-searched for additional studies. The search was designed with the aid of the following tools: the Systematic Review Accelerator (https://pubmed.ncbi.nlm.nih.gov/32004673/) and the Deduplicator (https://pubmed.ncbi.nlm.nih.gov/32004673/).

### Study Selection

**Figure 1.**
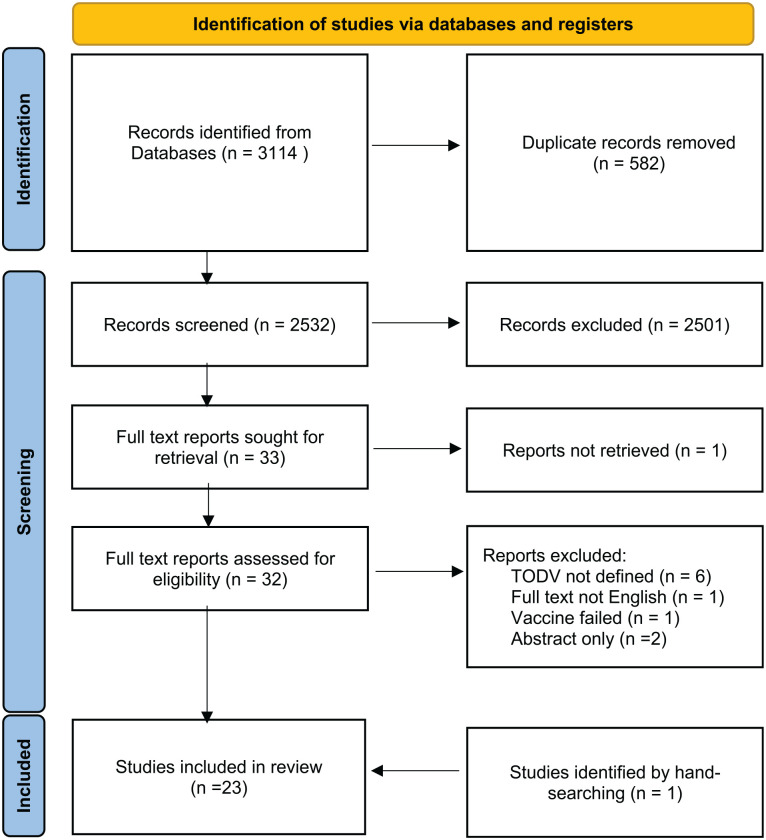
Screening and selection of eligible studies. Abbreviation: TODV = time of day of vaccination.

Two reviewers (CW and LR) screened the titles and abstracts of the papers retrieved by the search, and a third reviewer was consulted if the two assessments disagreed. There were no restrictions on age or TODV, nor the type of vaccination. Studies were included if they reported any immune or clinical outcome following vaccination at a defined time of day. Categorical definition, such as morning or evening, was included. Animal studies were excluded. Review papers, case studies, and conference abstracts with no primary data were excluded as were editorials and opinion pieces. Clinical trials, observational, cohort, and retrospective study designs were included regardless of randomization of vaccination time. The comparison was immune and clinical response to morning vaccination against vaccination at any other time of day, and the outcome defined as change in serology, immune cell numbers, phenotype or function, infection, or local or systemic adverse effects. Studies were selected if these outcomes were assessed at least once after the first or any subsequent dose of vaccine. Figure 1 summarizes the screening and the studies eliminated at full-text screening and reasons for exclusion are shown in Supplementary Table S1.

### Outcome Measurement and Data Extraction

Outcome variables were (1) antibody titer post vaccination, (2) seroconversion, (3) white blood cell phenotype and function, (4) self-reported adverse effects, (5) infection with the pathogen vaccinated against, or (6) hospitalization with disease vaccinated against. Data were extracted by two reviewers (EN and CW), and included information on the year of publication, study design, period and location, study population, type of vaccine(s) and intervention(s), outcome measures, results and conclusions was extracted from the included studies (Table 1). Risk of bias was assessed with the Risk Of Bias In Non-randomized Studies–of Interventions (ROBINS-I) tool for non-randomized studies as described (Sterne et al., 2016) and randomized studies were assessed with the Risk of Bias 2 (ROB2) tool (Sterne et al., 2019).

**Table 1.** Description of studies (*n* = 23) that investigated the effect of TODV and immune outcomes that were included in this review.

Study, Year, Country	Study Type Duration	Participants, Setting	*n*	Age (Years)	Female *n* (%)	Vaccine (Type)	Dose	TODV	Outcome	Confounding Factor Adjustments
**Hazan**, **2022, Israel**	Retrospective cohort December 2020-April 2022	Population sample, national health service	1.5 m	>12	1,095,633 (51.7)	COVID-19 (mRNA)	2	0800 h-1200 h 1200 h-1600 h 1600 h-2000 h	SARS-CoV-2 infection, ED visits, hospitalization	Sex, age, co-morbidity
**Phillips (i)**, **2008**, **United Kingdom**	Prospective trial Duration unknown	Students, primary care	75	22.9 (3.9) mean (SD)	41 (55)	Hepatitis A (Inactivated virus)	1	1000 h-1200 h 1600 h-1800 h	Hepatitis A anti-HAV antibodies	Sex, baseline antibodies
**Phillips (ii)**, **2008**, **United Kingdom**	Prospective trial Duration unknown	GP surgery patients, university	95	73.1 (5.5) mean (SD)	51 (57)	Influenza (Inactivated virus)	NI	0800 h-1100 h 1300 h-1600 h	Influenza HA antibodies	Sex, baseline antibodies, negative life events
**Long**, **2016**, **United Kingdom**	Cluster randomized trial 2011-2013	GP surgery patients, primary health care	276	<65	136 (49)	Influenza (Inactivated virus)	NI	0900 h-1100 h 1500 h-1700 h	Influenza HA antibodies	Baseline antibodies
**Matryba**, **2022**, Poland	Observational April 2021-June 2021	Medical students, university hospital	1324	23.3 (0.05) mean (SD)	959 (78)	COVID-19 (mRNA)	1 and 2	<1100 h >1500 h	SARS-CoV-2 anti-S1 IgG	None
**Jolliffe**, **2022**, **United Kingdom**	Retrospective cohort May 2020-October 2021	Population sample, public health services	9101	62 (57.1-69.9) median (IQR)	6414 (71)	COVID-19 (Viral vector, mRNA)	2	<1200 h 1200 h-1400 h 1400 h-1700 h >1700 h	SARS-CoV-2 anti-spike IgG, IgA, IgM	66 Sociodemographic, behavioral, clinical, pharmacological, nutritional factors
**Liu**, **2022**, China	Randomized trial October 2020-December 2021	Population sample, public health services	418	50-75	243 (62)	Influenza (Inactivated virus)	NI	0900 h-1100 h 1500 h-1700 h	Influenza HA antibodies	Stratification by age, gender
**Whittaker (i), 2022**, **United Kingdom**	Longitudinal observational Duration unknown	Students, university	75	22.9 (3.89) mean (SD)	41 (54)	Pneumococcal (Inactivated virus)	1	1000 h-1200 h vs 1600 h-1800 h	Pneumococcal IgG	Sex
**Whittaker (ii)**, **2022**, **United Kingdom**	Longitudinal observational Duration unknown	Parents, research participants	61	41.4 (5.31) mean (SD)	43 (70)	Pneumococcal (Inactivated virus)	1	Morning Afternoon	Pneumococcal IgG	Social support, sex
**Nachtigall**, **2022**, Germany	Retrospective observational May 2021-June 2021	Hospital employees, workplace	8375	18-61	6131 (74)	COVID-19 (Viral vector, mRNA)	1, 2, and 3	Morning Afternoon	Days off work, adverse events	Gender, age, vaccine type, dose
**Filippatos**, **2022**, Greece	Prospective observational January 2021-December 2021	Healthcare workers, university	468	48.3 (13.0) mean (SD)	361 (77)	COVID-19 (Viral vector, mRNA)	1, 2, and 3	0700 h-1100 h 1100 h-1500 h 1500 h-2200 h	SARS-CoV-2 total, neutralizing anti-RBD antibodies	Age
**Wang et al., 2022b**, **United Kingdom**	Retrospective observational December 2020-February 2021	Healthcare workers, hospital	2784	16-74	2302 (82)	COVID-19 (Viral vector, mRNA)	1	0700 h-1100 h 1100 h-1500 h 1500 h-2200 h	SARS-CoV-2 anti-spike, anti-nucleocapsid IgG	Vaccine type, age, sex, days post vaccination
**Langlois (i), 1995**, **United States**	Retrospective observational Summer 1984 and 1985	Company employees, clinic	98	45 (14.6) mean (SD)	NI	Influenza (Inactivated virus)	NI	Continuous 0900 h-1600 h	Influenza HA antibodies	Age, sex, race, infection, baseline titer
**Langlois (ii)**, **1996**, **United States**	Prospective observational Autumn 1985	Community, clinic	730	43.9 (0.9) mean (SD)	NI	Influenza (Inactivated virus)	NI	Continuous 0900 h-1500 h	Influenza HA antibodies	Age, sex, race
**Yamanaka**, **2022**, Japan	Retrospective observational 11 August 2021-27 August 2021	Staff and students, university	332	20-64	184 (55)	COVID-19 (mRNA)	1	Morning Afternoon	SARS-CoV-2 neutralizing and anti-nucleocapsid IgG	Days post vaccination, sex, age, allergy, medication, sleep duration
**Gottlob**, **2019**, Germany	Randomized trial 2015-2017	Premature infants, hospital	26	60 (1.09) days	15 (58)	Hexavalent primary (Inactivated virus)	1	0700 h-1000 h 1900 h-2200 h	Hexavalent vaccine antibodies	None
**Abbaspour**, **2022**, **United States**	Observational December 2020-April 2021	Employees, workplace	53,484	18-95	36,801 (73)	COVID-19 (mRNA)	1	0600 h-1100 h 2300 h-1600 h 1600 h-2200 h	Adverse events	Age, sex, ethnicity, history of allergy, epinephrine prescription
**Kurupati**, **2017**, **United States**	Retrospective observational 2011-2015	Community sample, university	139	30-40 and >65	93 (67)	Influenza (Inactivated virus)	NI	2000 h-1200 h 1200 h-1700 h	B-cell subsets, blood transcriptome, neutralizing IgG	None
**Karabay**, **2008**, Turkey	Randomized trial 6 months	Medical students, university	65	19-23	36 (57)	Hepatitis B (Recombinant)	3	0800 h-0830 h 1730 h-1800 h	Hepatitis B surface antigen, total core antigen antibodies	None
**Erber**, **2023**, Austria	Retrospective observational March 2021-April 2021	University staff, university	803	41.9 (11.9) mean (SD)	485 (60)	COVID-19 (Viral vector)	1	Continuous 0900 h-1600 h	SARS-CoV-2 anti-spike IgG	Baseline titer, sex, age
**de Bree**, **2020** Netherlands	Retrospective case control April 2017-June 2018	Healthy volunteers, university	52	26 median	33 (61)	BCG (Live attenuated)	1	0800 h-1200 h 1800 h-1830 h	PBMC cytokine production ex vivo	None, age, sex matched
**Zhang**, **2021**, China	Prospective cohort 2021-2022	Healthcare workers, hospital	63	26 (24-28) median (IQR)	37 (59)	COVID-19 (Inactivated virus)	1 and 2	0900 h-1100 h 1500 h-1700 h	B-cell subsets, SARS-CoV-2 neutralizing antibodies, ex vivo B and T-cell responses	Age, gender
**Bohn-Goldbaum**, **2022**, Australia	Retrospective observational January 2020-December 2020	Population sample, public health services	308,481	42.1 (27.1) mean (SD)	175,165 (58)	Influenza (Inactivated virus)	NI	Morning Evening	Adverse events	Age, sex, concomitant vaccine, vaccine type
**Feigin**,**1967**, **United States**	Controlled trial 11 days	Research participants, research setting	40	19-26	0%	Venezuelan equine encephalomyelitis (Live attenuated)	1	0800 h 2000 h	Virus-specific lymphocyte immunofluorescence	None
**Pollman**, **1988**, Germany	Retrospective observational 1985-1987	Healthcare workers, hospital	537	NI	318 (59.2)	Hepatitis B (Recombinant)	3	0730 h-0900 h 1300 h-1500 h	Anti-hepatitis B antibodies	Age, sex, season, body weight
**Lai**, **2023**, China	Randomized trial April 2021-May 2021	Population sample, research setting	503	33 (9) mean (SD)	318 (68)	COVID-19 (Inactivated virus)	2	0900 h-1100 h 1500 h-1700 h	SARS-CoV-2 neutralizing antibodies	Sex, age

Abbreviations: TODV = time of day of vaccination; BCG = Bacillus Calmette-Guérin; SARS-CoV-2 = severe acute respiratory syndrome coronavirus 2; mRNA = messenger ribonucleic acid; ED = emergency department; IgG = immunoglobulin G; IgA = immunoglobulin A; IgM = immunoglobulin M; SD = standard deviation; IQR = interquartile range; PBMC = peripheral blood mononuclear cell; RBD = receptor binding domain; GP = general practice; HAV = hepatitis A virus; HA = hemagglutination assay; NI = No Information.

### Data Analysis

The studies included in this review differed in terms of the disease vaccinated against, the type of vaccine (live, inactivated, mRNA), and the viral strains incorporated. Within those studies that did investigate the same vaccine, there was no consistency between the dose studied and the interval between doses, in which both factors were expected to affect the response to vaccination much more strongly than the TODV. Due to this heterogeneity, it was not considered appropriate to attempt a meta-analysis and a narrative synthesis approach was employed. The size of the effect of TODV relative to other factors affecting vaccination outcome was presented graphically where these data were available.

## Results

### Yield of Literature Search

The initial search yielded 3114 studies. Title and abstract searches resulted in exclusion of 2501 records, and 582 duplicates were removed, leaving 33 studies for full-text review. A further exclusion of 11 studies were done at this stage and one study was retrieved through hand-searching (see Suppl. Table S1 for details). A total of 23 studies met all criteria and were selected for inclusion in the systematic review (Figure 1). Details of these studies are given in Table 1.

The 23 eligible studies were published between 1967 and 2023 and reported results of studies carried out in 12 countries including United States (*n* = 4), United Kingdom (*n* = 5), China (*n* = 3), Germany (*n* = 3), Australia (*n* = 1), and other European countries (*n* = 5). There were 388,714 participants (range 26-308,481; mean ± SD 16,196 ± 36,208) in 22 studies, with one study (Hazan et al., 2023) considered an outlier in terms of numbers of participants (*n* = 1,515,754).

The study settings were mostly health care or research based: hospital/clinic (*n* = 8), public health service (*n* = 6), and university/research institute (*n* = 9). There were 5 randomized controlled trials, 8 retrospective and 8 prospective observational studies, and 2 non-randomized trials. The majority of the studies investigated the effects of TODV of severe acute respiratory syndrome coronavirus 2 (SARS-CoV-2; *n* = 11) or influenza (*n* = 7) vaccines, and the remainder investigated *Bacillus Calmette-Guérin* (BCG; *n* = 1), hepatitis (*n* = 3), pneumococcus (*n* = 1), hexavalent (*n* = 1), and encephalitis (*n* = 1) vaccination (Table 1).

### Participant Demography

Most of the eligible studies recruited participants from the community (45%) and healthcare workers (23%) and the remaining studies recruited students (18%) and employees (9%). The age range of participants was 12-74 years, with 2 studies including children and 6 studies including people aged over 60 years only (Table 1). The majority of the studies had a higher proportion of female participants and 6 studies had more than 70% female participants (Erber et al., 2023; Filippatos et al., 2022; Long et al., 2016; Matryba et al., 2022; Nachtigall et al., 2022; Phillips et al., 2008; Table 1). One study had 100% male participants (Feigin et al., 1967). Some studies reported that women were more likely to participate in studies of TODV, more likely to report adverse reactions to vaccination (Nachtigall et al., 2022), and more likely to have a higher antibody titer post vaccination than men (Nachtigall et al., 2022; Wang et al., 2022b).

There was low or poorly documented ethnic diversity in the 23 studies; 6 studies gave details of the ethnicity of participants and the majority of their participants were White (Abbaspour et al., 2022; Jolliffe et al., 2022; Langlois et al., 1995; Matryba et al., 2022; Phillips et al., 2008; Whittaker et al., 2022). Just one study included ethnicity as a covariable in a multivariable analysis of the association between TODV and outcome (Jolliffe et al., 2022).

Work status could be implied from studies in the workplace (university, hospital, etc) (9/23 studies), but only one study electively accounted for this factor (Jolliffe et al., 2022). Three studies of people of working age accounted for shiftwork through exclusion or adjustment (Erber et al., 2023; Matryba et al., 2022; Yamanaka et al., 2022).

There were some reports of associations between demographic factors and the TODV.

In one study, younger people tended to select either early morning or late afternoon appointments (Kurupati et al., 2017). In a UK population-wide study, people vaccinated against COVID-19 in the morning tended to have fewer co-morbidities (Jolliffe et al., 2022), while in a similar study in Israel, the participants vaccinated in the morning tended to have more co-morbidities and to be older (Hazan et al., 2023). Just one study considered the effect of chronotype and reported no association with vaccination outcome (Matryba et al., 2022). One study considered circadian timing; Bohn-Goldbaum et al. (2022) reported no association between the interval between vaccination and wake time and adverse events post vaccination. The associations between TODV and outcome were thought to be stronger in aged participants in two studies (Kurupati et al., 2017; Liu et al., 2022), although there was considerable variability among vaccine types. One study reported that the effects of TODV were stronger in women (Liu et al., 2022), and another in men (Erber et al., 2023).

### Vaccination History and Baseline Immune Status

Immune status at baseline was accounted for in most studies by measuring antibody titers before vaccination, and/or by reporting previous vaccination and infection history but 6 studies did not assess prior vaccination or infection status at baseline (Abbaspour et al., 2022; Bohn-Goldbaum et al., 2022; Kurupati et al., 2017; Langlois et al., 1995; Long et al., 2016; Phillips et al., 2008; Zhang et al., 2021; Suppl. Table S2). Some studies reported that there was already a significant difference in immune status (antibody titer or B-cell subsets) between morning and afternoon/evening groups before the vaccine was administered (Kurupati et al., 2017; Long et al., 2016; Zhang et al., 2021).

There was no consistency in the treatment of participants that remained seronegative after vaccination between studies; some studies performed sub-group analysis (Jolliffe et al., 2022; Matryba et al., 2022), but most studies gave no information about how data from participants that did not respond to vaccination were analyzed. The dose of vaccine used varied widely between studies; immune response to the first vaccine dose was reported in 10 studies, to the second in 3 studies, and 4 studies reported data on combinations of response to multiple doses of vaccine. There was no information on the dose administered in 6 studies (Table 1). Most study durations spanned more than 6 months (14 ± 11 months; mean ± SD), and 6 studies were completed over 2 years or more (Hazan et al., 2023; Kurupati et al., 2017; Langlois et al., 1995; Long et al., 2016; Phillips et al., 2008; Pollmann and Pollmann, 1988).

### Definition and Allocation of TODV

**Figure 2.**
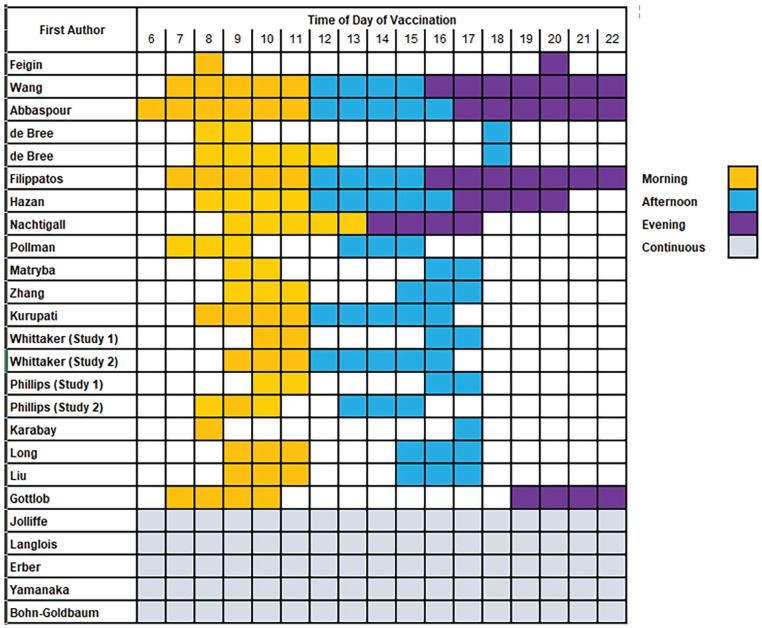
Summary of time intervals of vaccinations in selected studies.

There was considerable variation in the definition of TODV (Figure 2); 3 studies reported TODV as a continuous variable, 2 as a binary or categorical am/pm or morning/afternoon variable, and the remainder reported morning and afternoon/evening as a time interval defined by clinic times or by unjustified decisions (Table 1). Across all studies, the times of morning vaccination ranged between 0600 h and 1300 h, afternoon between 1200 h and 1800 h, and evening between 1600 h and 2200 h (Figure 2). Some studies that assessed the effect of TODV on outcome at more than two timepoints reported that the relationship was non-linear (Filippatos et al., 2022; Wang et al., 2022b), or in the case of continuous measurements, reached a peak and trough within 12 h (Erber et al., 2023; Hazan et al., 2023; Langlois et al., 1995).

There were 5 studies that randomized participants to receive either morning or afternoon vaccination (Gottlob et al., 2019; Karabay et al., 2008; Lai et al., 2023; Long et al., 2016; Zhang et al., 2021), 3 studies allowed self-selected TODV (Phillips et al., 2008; Whittaker et al., 2022; Yamanaka et al., 2022), and in 2 studies TODV was allocated by an administrator (Erber et al., 2023; Zhang et al., 2021). In all other studies there was no information on how the TODV was allocated (Suppl. Table S2). In 2 of the 5 randomized studies (Phillips et al., 2008; Whittaker et al., 2022), 30% of participants were allowed to switch intervention (TODV) after allocation which invalidated the randomization procedure. In all other studies, there was no information about whether switching between interventions (i.e., between morning and evening TODV) was permitted.

### Immune Outcomes

The immune outcome considered (see Table 1) was most commonly antibody titer post vaccination; 2 studies considered seropositivity and 7 reported the number of adverse events post vaccination. Infection was the outcome variable in 3 studies. White blood cell phenotypes and function were less commonly assessed, as reported by 4 studies (Table 1).

### Timing of Post-vaccination Follow-up

The interval of time elapsed between vaccination and follow-up differed between participants in most studies, as well as between studies (Suppl. Table S3). In studies that compared the response to vaccination against baseline measurements, 5 matched the circadian timing of the baseline and post-vaccination blood sample, the time of baseline and post-vaccination samples were misaligned in 5 studies, and in remaining cases the temporal alignment between baseline and follow-up samples was not clear (Suppl. Table S3). In 2 studies, the timing of the post-vaccination blood sample was thought to affect the significance of the TODV effect on outcome (de Bree et al., 2020; Kurupati et al., 2017).

### Effect of TODV

The data reported on the effect of TODV on immune and clinical outcomes are shown in Supplementary Table S2, and the range and times of day investigated in each study are shown in Figure 2. The heterogeneity between studies in the types of vaccine, the TODV, and the time interval between vaccination and follow-up that precluded meaningful meta-analysis and individual data from each study is given for comparison (Suppl. Table S2). Over 40% of studies (10/23) did not detect any beneficial TODV and 3 studies reported significant non-linear associations between vaccination outcome and TODV. The optimum TODV was concluded to be afternoon (5 studies), morning (5 studies), morning and afternoon (1 study), midday (1 study), and morning or late afternoon (1 study) with the remaining 10 studies reporting no effect.

**Figure 3.**
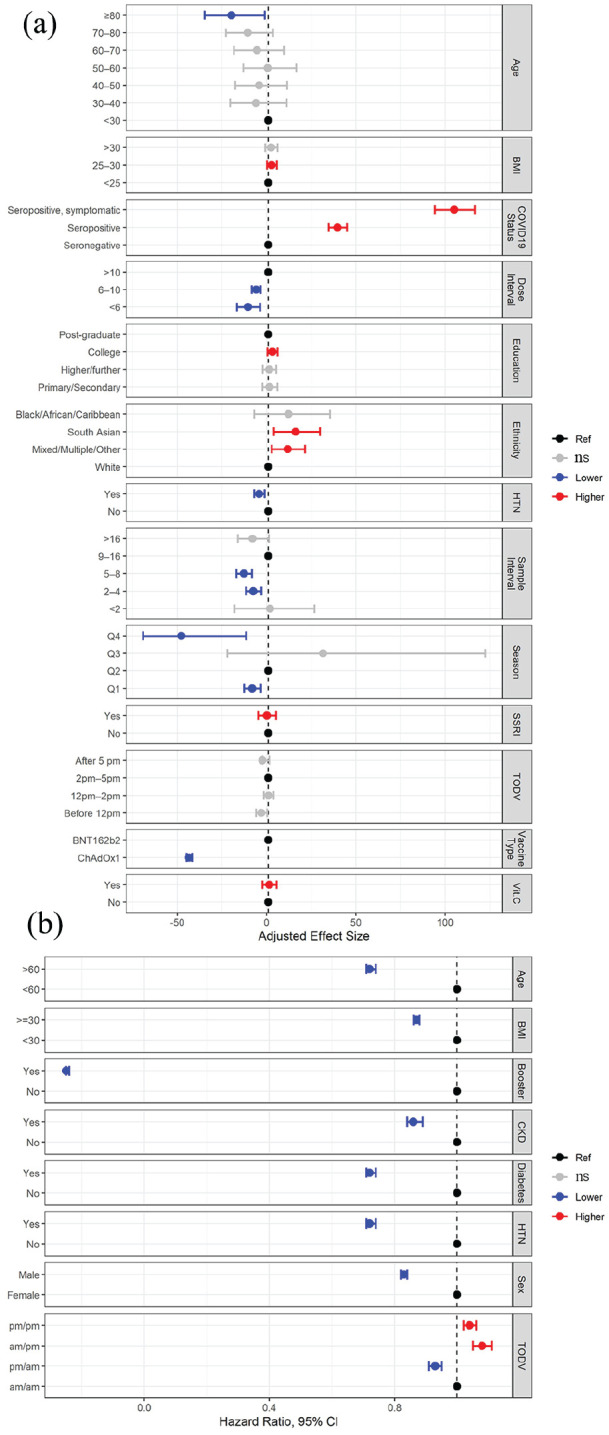
Summary of results of two studies that compared the effect size of TODV against other factors affecting response to vaccination. (a) Multivariable analyses reported by Jolliffe et al. (2022) showed a small (non-significant) effect of TODV on vaccination outcome (change in antibody titer) relative to other factors. (b) The effect of demographic and health-related factors, including different permutations of TODV on the risk of breakthrough infection after vaccination as reported by Hazan et al. (2023). Abbreviations: HTN = hypertension; ns = not significant; TODV = time of day of vaccination; CKD = chronic kidney disease; CI = confidence interval; BMI = body mass index; SSRI = Selective serotonin reuptake inhibitors; Vit.C = Vitamin C.

Of the studies that reported an association between TODV and outcome of vaccination, 3 presented data that could be used to estimate the size of this effect (Erber et al., 2023; Hazan et al., 2023; Zhang et al., 2021). In one study, morning or afternoon vaccination was associated with decreased probability of infection compared to evening vaccination, 0.95 (0.94-0.96) and 0.92 (0.91-0.93), odds ratio (OR; 95% confidence interval [CI]; *n* = 1,515,754) for morning and afternoon, respectively (Hazan et al., 2023). Zhang et al. (2021) reported that antibody titers were significantly higher in healthcare workers (*n* = 67) after morning vaccination, with the difference being 14.84 (7.37-24.15) AU/ml, median (interquartile range). Erber et al. (2023) reported increased probability of lower antibody titers after vaccination at 1200 h-1300 h (1.45 (1.12-1.87), OR (95% CI; *n* = 803) compared to 0900 h-1000 h. The remaining studies either report non-significant effects or did not present data on effect size. Data from two studies that reported the effect size of TODV relative to other predictors of vaccination outcome are presented graphically (Figure 3).

### Risk of Bias

Risk of bias in randomized controlled trials was assessed using the ROB2 tool (Sterne et al., 2019) and in non-randomized trials with the ROBINS-I tool (Sterne et al., 2016). The risk of bias for all studies ranged from moderate to critical (Tables 2 and 3) with most studies scoring poorly in the domains of baseline confounding and measurement of outcomes. The main issues identified with baseline confounding were failure to account for existing immune status prior to vaccination, co-morbidity, or the underlying circadian rhythmicity of immune function. All but two studies (Lai et al., 2023; Matryba et al., 2022) were considered to be biased by their failure to assess or account for individual chronotype. Most studies with self-selected TODV did not account for behavioral parameters that might determine the selected or allocated time of day, such as work status/role or geographic location. Bias in the measurement of outcomes was considered to be moderate to serious if the risk of allocation to an intervention (e.g., morning vaccination) was related to the immune status. For example, healthy working people might select TODV outside office hours, and be more likely to have good vaccination outcomes. Studies were considered to be biased in outcome measurements if there were sequential hypothesis testing of differences between timepoints, related immune outcomes, and vaccine viral strains without correction for multiple comparisons. The classification of intervention was considered to be a source of bias where the definition of TODV was unclear, or not consistent between participants. In most cases, these sources of bias were acknowledged by the study authors in their discussion and the scores allocated reflect the complexities of studying human response to vaccination.

**Table 2.** Risk of bias in the included studies assessed by the ROBINS-I tool.

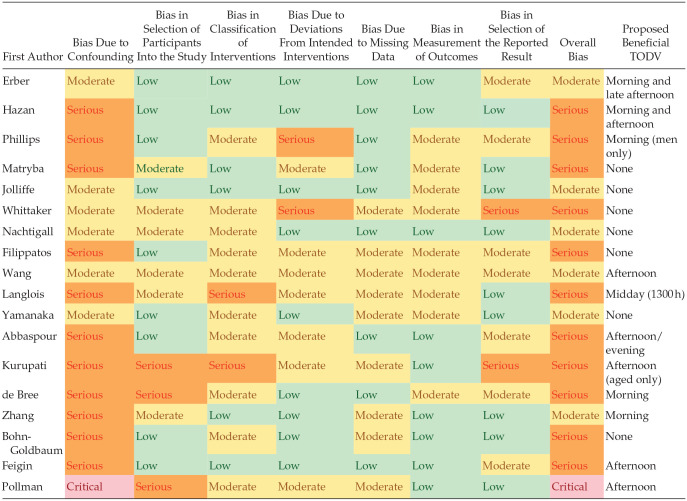								

Abbreviations: TODV = time of day of vaccination; ROBINS-I = Risk Of Bias In Non-randomized Studies–of Interventions.

**Table 3.** Risk of bias in the included studies assessed by the ROB2 tool.

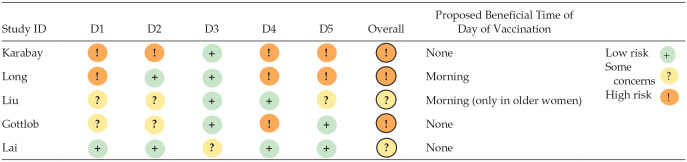								

Abbreviations: D1 = randomization process; D2 = deviations from the intended interventions; D3 = missing outcome data; D4 = measurement of the outcome; D5 = selection of the reported result; ROB2 = Risk of Bias 2.

## Discussion

This systematic review of 23 studies of circadian timing of vaccination revealed that while some studies reported an effect of TODV, there is insufficient overall evidence that administration of vaccines at different times of day affects immune outcomes. Generalizing the findings of the included studies was challenging due to their heterogeneity and an overall effect and potential clinical benefit of vaccination at different times of day are not excluded.

The ROBINS-I tool was applied to assess the risk of bias in the non-randomized studies but the diversity of study designs and populations included makes comparison of bias between the included studies using this study challenging and subjective. Nevertheless, the tool did provide a quantitative framework that helped assess the sources of bias and how they were addressed in each study.

The majority of studies exhibited bias ranging from moderate to critical and there was considerable heterogeneity between studies in terms of vaccine type, dose, interval between vaccination and follow-up, and outcome variables. Most studies had small sample sizes and there were no large-scale randomized controlled studies. There were two large population-level studies but these were confounded by poor definition of the factors that determined allocation to an intervention (TODV; Jolliffe et al., 2022) and by the potential effects of social restriction during the COVID-19 pandemic on the outcome variable (infection; Hazan et al., 2023). Participant demography was sometimes related to the TODV. In one study, younger people tended to select either early morning or late afternoon appointments (Kurupati et al., 2017) possibly to accommodate work times. The studies included in this review varied extensively in their management of factors known to strongly determine response to vaccination such as type of vaccine, baseline immune status, co-morbidity, age, interval between vaccination and follow-up, and interval between doses (Lange et al., 2003; Tsang et al., 2014; Zimmermann and Curtis, 2019). It follows that their conclusions about the optimum TODV also vary, with some proposing morning, afternoon, evening, and midday, and the majority failing to find evidence to support any association between TODV and outcome.

In some studies, the TODV was self-selected or could be rescheduled by the participant, which favors alignment of TODV with individual circadian rhythms, so that people with morning chronotypes might present for vaccination earlier in the day. It is well established that people with a daily preference for activities later in the day are likely to have more co-morbidities (Knutson and von Schantz, 2018) and harmful lifestyle behaviors (smoking, screen use, poor diet, low physical activity; Patterson et al., 2016), all factors that might affect vaccination outcome and confound detection of any effect of circadian rhythms in immune function (Dobaño et al., 2022; Karachaliou et al., 2022; Moncunill et al., 2022). In addition to such confounding by chronotype, self-selection allows the TODV to be inadvertently associated with vaccination outcome by demographic factors. Working status is one such factor since people in full-time employment are more likely to be younger, healthier, and might select appointments at lunchtime vaccination or times outside working hours (0900 h-1700 h), but only one study electively accounted for this factor (Jolliffe et al., 2022).

Shiftwork adds a further level of complexity to studies of TODV in workers, by affecting both the outcome (response to vaccination) and the likelihood of morning vaccination. Shift workers are likely to have short sleep durations (Kecklund and Axelsson, 2016), likely to have more co-morbidities (Kecklund and Axelsson, 2016), and likely to smoke (Patterson et al., 2016) compared to day workers, all factors that affect vaccination outcome. The work patterns and disrupted circadian rhythms of shift workers might determine their TODV where self-selection or rescheduling of vaccination time was permitted. Regardless of any effect of shiftwork on the TODV (intervention) or response to vaccination (outcome), the disrupted circadian rhythms that these work patterns induce would affect the position of the optimal window for vaccination within a day should one exist. Some studies of people of working age included in this review accounted for these possibilities by excluding or adjusting for shiftwork (Erber et al., 2023; Matryba et al., 2022; Yamanaka et al., 2022) but most did not consider shiftwork at all. There is evidence that sleep deprivation in the days before and after vaccination can affect the immune response (Lange et al., 2003, 2011; Spiegel et al., 2023) and it is possible that increasing homeostatic sleep pressure through the day and variation in sleep deprivation between participants could confound effects of TODV in studies that did not control for this factor.

Most investigations of the association between TODV and vaccination outcome are derived from studies of healthcare workers, students, and university staff. The demography of these cohorts presents factors that affect TODV such as age, work schedule, access to vaccination, and disrupted circadian rhythms from shiftwork or student lifestyles. Studying the TODV in frontline healthcare workers is further affected by their increased risks of exposure to infectious disease that would affect their baseline immunity and vulnerability to breakthrough infection, as well as boost antibody levels if natural challenge occurred post vaccination. The TODV of health care workers could be linked to their role in the health care setting if selected to accommodate shift patterns, or if blocks of vaccination appointment times were allocated to those most at risk of exposure. Most of the studies included in this review involved health care workers and/or medical students and their conclusions should be reproduced in a population sample.

Many demographic factors could confound detection of an endogenous circadian rhythm in response to vaccination through their effects on both TODV and vaccination outcome. This was illustrated in a study of a SARS-CoV-2 prophylactic intervention (BCG vaccination) where participants in the control group were significantly more likely to develop a COVID-19 infection after being administered a placebo (saline injection) in the morning compared to the afternoon (Föhse et al., 2023). The factors that influence individual allocation of TODV are multi-factorial, often related to vaccination outcome and are probably only controlled through randomized population-level studies.

Most of the studies included in this review spanned several months or even years (Hazan et al., 2023; Kurupati et al., 2017; Long et al., 2016; Pollmann and Pollmann, 1988), so that the season of vaccination and the interval between vaccination and follow-up differed between participants and studies. This variation introduces bias due to endogenous seasonal variation in immune function, variation in the prevalence of circulating viral strains, and different viral strains included in seasonal vaccines. One study reported that the season had a significant effect on the antibody response to vaccination, while the TODV was not significant (Jolliffe et al., 2022). There are well-established relationships between season and viral infection, and similar associations with vaccination are worthy of investigation.

Prior infection, exposure, and vaccination history strongly affect response to vaccination (Moncunill et al., 2022; Wu et al., 2022; Zimmermann and Curtis, 2019) but not all studies accounted for these factors by assessing antigen-specific immune status at baseline. Circadian regulation of memory and adaptive immune responses to vaccination could be different, and antigen-specific immune status at baseline should be consistent between participants in studies of the TODV. In addition to antigen-specific immunity, previous vaccination against unrelated pathogens could affect vaccine response through “trained immunity,” where vaccination induces heterologous protection beyond the target disease (Benn et al., 2013). The interval between vaccination and follow-up sampling could further confound detection of an effect of TODV when antibody titer is taken to represent the response to vaccination; this interval differed between participants as well as between studies included in this review.

A common source of bias occurred when baseline and follow-up samples were not collected at the same time of day, making putative changes related to TODV vulnerable to the effects of circadian rhythmicity in the outcome variable. Stable secretion of antibodies over 24 h was assumed by most of the studies included in this review which seems at odds with the overall hypothesis that endogenous circadian regulation of leucocyte function could affect response to vaccination. Rhythmicity of outcome variables at baseline and follow-up could both affect detection of an effect of TODV but no study adequately controlled or adjusted for this complexity in clock-mediated regulation of immune function. Indeed, several studies reported a time-of-day effect on antibody levels at baseline (Kurupati et al., 2017; Long et al., 2016; Zhang et al., 2021), which suggests that either distinct immune phenotypes tend to be vaccinated at certain times of day or that circadian variation in immune function is evident in the outcome variable at baseline. This circadian variation could be innate, as reported in animals (Cermakian et al., 2022), or secondary to masking by daily behavioral (e.g., work times) or physiological ultradian rhythms (e.g., cortisol).

The influence of circadian variation in antibody secretion after vaccination can only be resolved by sequential blood sampling over 24 h at baseline, and at post-vaccination follow-up. There have been no studies to our knowledge that have taken this approach in humans, or even in mammals, but one study in fish demonstrated circadian rhythms in antibody secretion that were disrupted by vaccination (Guerra-Santos et al., 2018). While there is compelling evidence for circadian regulation of immune function in animals (Edgar et al., 2016; Fortier et al., 2011; Silver et al., 2012) and daily variation in some human immune parameters (Born et al., 1997; Wyse et al., 2021), it remains unclear whether human antibody production shows daily rhythmicity (Wyse et al., 2021). The effects of vaccination on such rhythms (if they exist) are also unknown, and all these issues must be resolved before antibody titer can be used as a proxy measure of vaccine effectiveness in chronobiological studies. Animal studies of the effects of TODV have focused on innate immunity, and the mechanisms through which TODV might affect long-term immune responses such as T-cell differentiation and B-lymphocyte maturation are unclear (Hemmers and Rudensky, 2015). The response to mRNA, vector, and inactivated vaccines is elicited through different immune pathways that might be subject to varying degrees of circadian regulation. Consequently, the effect of TODV could be dependent on the type of vaccine, and this could account for some of the variation between the studies included in this review. Further studies are required to understand the circadian regulation of different immune mechanisms and their implication for chronovaccination.

It is of interest that most of the studies that assessed the effects of TODV at more than two timepoints reported associations with outcome that were non-linear, with a peak and trough within a 12-h period, suggesting an ultradian rather than a circadian pattern (Hazan et al., 2023; Langlois et al., 1995; Wang et al., 2022b). Such non-linear relationships would be missed by the majority of studies that assessed the effects of TODV at two timepoints. Previous studies of clock-regulated immune function in animal models and humans report oscillation over 24 h (Curtis et al., 2014; Labrecque and Cermakian, 2015; Wang et al., 2022a), and the ultradian patterns reported by studies in this review suggest that the circadian clock is not the predominant driver of the TODV effect they report. Nevertheless, endogenous timing is not excluded; there is increasing evidence supporting the existence of 12-h innate oscillators that are independent of the circadian clock (Zhu and Liu, 2023). In fact, autonomous ultradian rhythms with a 12-h period have been reported in the expression of mammalian genes involved in immune regulation, *Rela*, *Nfkb1*, and *Tnfaip3* (Pan et al., 2020). The differentiation and egress of hematopoietic stem and progenitor cells showed daily fluctuations that followed two daily peaks related to light and dark signals (Golan et al., 2018), although an endogenous origin for these patterns was not established. Rhythms with a period of 12 h arose earlier in evolution than circadian rhythms, driven by the requirements of ancient, ocean-dwelling creatures to entrain to the 12-h rhythms of the tide rather than the 24-h light-dark cycle that would later drive evolution of the circadian clock in terrestrial animals. Their significance in mammals is poorly understood, and their contribution to ultradian patterns in the response to TODV is purely speculative. It is more likely that ultradian patterns of response to vaccination are driven by human daily behavior patterns that affect the allocation of TODV, whereby specific demographic groups attend for vaccination at times determined by the ultradian timing of work or social commitments, commute time, occupation, clinic opening times, or distance of residence from vaccination centers. It is also possible that ultradian patterns in physiology generated by eating, stress or exercise times, or endogenous cortisol ultradian rhythms could affect response to vaccination.

The global vaccination program implemented during the COVID-19 pandemic presented an opportunity to investigate the importance of TODV, but one that was critically confounded by the systems through which TODV was allocated, and the extraordinary lifestyle changes imposed during the pandemic. The world-wide restrictions on social mixing implemented to control transmission of SARS-CoV-2 (e.g., social distancing, remote working, cocooning, lock-down) could affect conclusions about TODV. For example, the risk of exposure throughout the pandemic was highly variable between participants; both their TODV and their vulnerability to infection and humoral response to vaccination could have been affected by occupation, prevailing control measure, waves of infection, and SARS-CoV-2 variants. In support of this, the factors usually associated with susceptibility to infection (age, co-morbidity, obesity) were protective in a population-level study of the TODV during the pandemic (Figure 3b; Hazan et al., 2023), suggesting that the outcome measure (infection) was affected by social restriction of vulnerable people.

There was one randomized controlled study of TODV during the pandemic (that reported no effect; Lai et al., 2023), but the factors controlling allocation of morning or afternoon vaccination in the other studies during the pandemic were self-selected or unclear. In many cases, TODV might have been driven by vulnerability to infection, so that health care workers, older people, or people with co-morbidities had preferential access to appointments. Such allocation of the TODV by administrative or demographic factors (e.g., vulnerability, occupation, age, area of residence) or by self-selection could seriously confound detection of circadian rhythms in the response to vaccination. An ultradian association was reported between the TODV and the likelihood of self-reporting COVID-19 infection (positive polymerase chain reaction test) after vaccination in a large (*n* ~1.5 m) population sample during the pandemic (Hazan et al., 2023). The social restriction measures imposed during the pandemic caused variability in post-vaccination exposure to the virus between participants, and a self-reported infection outcome variable is compromised by the fact that the majority of post-vaccination infections are asymptomatic (North et al., 2022), and were likely to be missed.

### Future Research

There are many unanswered questions that must be addressed before consideration of the TODV in the clinical setting. Circadian regulation of vaccination outcome measures such as antibody titers must be further understood in animal models, and their relationship with disease resistance established for all vaccines. Randomized trials at population level are essential to accommodate many demographic and environmental factors that affect both TODV and vaccination outcome in humans. The population-level studies included in this review that provided quantitative data on TODV report small effect sizes that suggest that sample sizes of several thousand participants should be recruited for future studies of TODV (Hazan et al., 2023; Jolliffe et al., 2022; Lai et al., 2023; Liu et al., 2022) although it must also be remembered that some studies detected statistically significant effects in much smaller samples of student or healthcare worker cohorts (e.g., Zhang et al., 2021, *n* = 62; Erber et al., 2023, *n* = 803). The advantage of large population-level studies is their power to adjust for the multiple demographic and lifestyle factors that might otherwise confound detection of an effect of TODV. Furthermore, investigation of the causal effects of daily variation in the response to vaccination will be facilitated by the availability of big datasets with rich individual-level information on health and lifestyle combined with advanced statistical and machine learning techniques. It should be considered that such population-level studies would be costly as stand-alone endeavors but could easily be incorporated into clinical trials of vaccination, where the onus is on the vaccine producers to demonstrate that effectiveness does not depend on the TODV. At a mechanistic level, future research should apply free-running protocols to establish whether circadian rhythms in human immune function truly reflect endogenous clock-mediated oscillation or are secondary to other features of human behavior and lifestyle that vary over 24 h. Studies that include vaccination times that extend further into the night (i.e., after 2100 h) would also be informative with respect to the role of the circadian clock in mediating time-dependent variability in the response to vaccination. Future research should also focus on the development of a simple method for assessment of human circadian phase that will allow endogenous daily variation in immunity to be linked to therapeutic benefit.

As a population as well as an individual prophylactic intervention, the benefit of time-dependent vaccination must be sufficiently great to justify its disruptive effect on the delivery of vaccination programs. Manipulation of the TODV or “chronovaccination” is an intervention proposed to target those that respond poorly to vaccination such as the aged or immunocompromised (Otasowie et al., 2022), yet most information available is from studies in students and healthcare workers. Further work should address this by studying the implications of the TODV in these groups whose compromised immune and circadian function might make their response to TODV quite different to that of healthy people.

### Strengths and Limitations

The principal strength of this review is our critical appraisal of all currently available data on the effect of TODV on vaccination outcome using an approach that adhered to recommended quality standards for conducting systematic reviews including a comprehensive search strategy and risk of bias assessment. This study also has limitations. The majority of the studies included had observational, retrospective study designs and in most cases, the factors controlling allocation to the intervention group (morning vaccination) were unknown. We did not include studies only available as abstracts, which might have excluded emerging evidence. We only included studies published in English which may have excluded relevant studies. Comparison between studies was difficult due to the heterogeneity in vaccine types, outcome variables, and study design, and this precluded meta-analysis.

It is a limitation that cross-sectional changes in antibody titer were used to quantify response to vaccination in most of the studies in this review rather than more objective methods for assessment of vaccine effectiveness such as randomized, placebo-controlled, double-blind trials. There is evidence to support the use of antibody titers as surrogate markers of efficacy for COVID-19 (Corbett et al., 2021) and influenza (Laurie et al., 2015) vaccines but these tests do not reflect cellular immunity nor the influence of other factors that might affect resistance to disease such as pre-existing immunity. The use of changes in antibody titer as a continuous outcome variable implies a direct, quantitative relationship between disease resistance and the proportional change in post-vaccination titer, which may not be justified. Future studies should assess the impact of TODV on effectiveness of vaccination in preventing infection or clinical disease to support findings from proxy measures of efficacy such as changes in antibody titer.

## Conclusions

At a population level, the efficacy of vaccination is compromised by vaccine hesitancy, a refusal to access vaccines due to complacency, lack of confidence, or inconvenience. Vaccine hesitancy is identified by the WHO as one of the 10 threats to global health (WHO, 2022) and its rise threatens to reverse progress made in eliminating infectious diseases such as measles, polio, and human papillomavirus. The Strategic Advisory Group of Experts on immunization which advises the WHO on vaccination strategies reported convenience, including access to vaccination at an appropriate time and place, to be one of the 3 main factors that influences vaccine uptake (WHO, 2022) which underlines the importance of accurate research and communication of the clinical significance of the TODV.

Circadian timing mechanisms regulate most aspects of human physiology, and response to vaccination is not likely to be an exception given existing evidence for daily variability in other aspects of human immune function (Born et al., 1997; Wyse et al., 2021). Furthermore, studies in mouse models provide compelling evidence that TODV can affect susceptibility to vaccination (Ince et al., 2023; Nobis et al., 2019), and mechanisms fundamental to adaptive immunity weeks after the initial challenge (Fortier et al., 2011; Ince et al., 2023; Silver et al., 2012; Suzuki et al., 2016). Nevertheless, mouse models poorly represent the circadian response to vaccination in humans because they live in a pathogen-depleted environment, they lack pineal melatonin, they are nocturnal, and they are not subject to the same daily variation in environmental challenges as humans. In further contrast to mice, relationships between the TODV and outcome in humans could be mediated by endogenous timing mechanisms in combination with environmental factors that also vary by time- of day (work, mealtimes, commuting, stress) and randomized controlled studies that control for these factors are required to support recommendations about TODV. Animal studies and prior evidence for circadian regulation of the human immune system provide mechanistic support for an effect of TODV on vaccination outcome that justifies consideration of TODV in future studies regardless of the uncertainty of current evidence. Chronovaccination could potentially improve response to vaccination in individuals and at population level, and the TODV should be considered in future studies of vaccine effectiveness. This review has identified multiple confounding factors that bias current evidence, as well as highlighted factors that should be considered in future studies.

## Supplemental Material

sj-docx-1-jbr-10.1177_07487304241232447 – Supplemental material for Circadian Variation in the Response to Vaccination: A Systematic Review and Evidence Appraisal Supplemental material, sj-docx-1-jbr-10.1177_07487304241232447 for Circadian Variation in the Response to Vaccination: A Systematic Review and Evidence Appraisal by Cathy A. Wyse, Laura M. Rudderham, Enya A. Nordon, Louise M. Ince, Andrew N. Coogan and Lorna M. Lopez in Journal of Biological Rhythms
