# Supplementary material for: Circadian Variation in the Response to Vaccination: A Systematic Review and Evidence Appraisal
Source: J Biol Rhythms. 2024 Mar 8;39(3):219–36. doi: 10.1177/07487304241232447 (PMC11141079; doi:10.1177/07487304241232447)
Supplement: sj-docx-1-jbr-10.1177_07487304241232447 – Supplemental material for Circadian Variation in the Response to Vaccination: A Systematic Review and Evidence Appraisal [file sj-docx-1-jbr-10.1177_07487304241232447.docx]

**Circadian Variation in the Response to Vaccination: A Systematic Review and Evidence Appraisal**

Wyse CA*^1^, Rudderham LM^1^_,_ Nordon EA^1^, Ince LM^2^, Coogan AN^3^ and Lopez LM^1^

^1^Kathleen Lonsdale Institute for Human Health Research and the Department of Biology

Maynooth University, Kildare, Ireland

^2^Division of Pharmacology and Toxicology, College of Pharmacy, University of Texas at Austin, Texas, USA

^3^Kathleen Lonsdale Institute for Human Health Research and the Department of Psychology, Maynooth University, Kildare, Ireland

**Supplementary Information 1 - Search Strategies**

**MEDLINE/OVID Search Criteria**

1 _exp Circadian Rhythm/ or circadian.mp.

2 _evening*.ti,ab.

3 _morning*.ti,ab.

4 _daytime.ti,ab.

5 _afternoon.ti,ab.

6 _night.ti,ab.

7 _nighttime.ti,ab.

8 _time of day.ti.ab.

9. _diurnal.ti.ab

10 _immuniz*.mp.

11 _exp Vaccination/

12 _exp Vaccines/

13 _vaccin*.ti,ab.

14 or/10-13

15 _animals/ not humans/

16 or/1-9

17 _16 and 14

18 _17 not 15

**PUBMED Search (23/02/23)**

1 Circadian Rhythm/ OR circadian

2 evening* [Title/Abstract]

3 morning* [Title/Abstract]

4 daytime [Title/Abstract]

5 afternoon [Title/Abstract]

6 night [Title/Abstract]

7 nighttime [Title/Abstract]

8 “time of day” [Title/Abstract]

9 diurnal [Title/Abstract]

10 immuniz* [Title/Abstract]

11 Vaccination/

12 Vaccines/

13 vaccin* [Title/Abstract]

14 #10 OR #11 OR #12 OR #13

15 animals/ NOT humans/

16 or/1-9

17 16 and 14

18 17 not 15

**Cochrane Clinical Trials Search (24/02/23)**

ID Search Hits

#1 circadian

#2 MeSH descriptor: [Circadian Clocks] explode all trees

#3 evening*

#4 morning*

#5 daytime

#6 afternoon

#7 night

#8 nighttime

#9 "time of day"

#10 immuniz*

#11 MeSH descriptor: [Vaccines] explode all trees

#12 MeSH descriptor: [Vaccination] explode all trees

#13 vaccin*

#14 #10 OR #11 OR #12 OR #13

#15 MeSH descriptor: [Animals] explode all trees

#16 MeSH descriptor: [Humans] explode all trees

#17 #15 NOT #16

#18 #1 OR #2 OR #3 OR #4 OR #5 OR #6 OR #7 OR #8 OR #9

#19 #18 AND #14

#20 #19 NOT #17

**EMBASE (24/02/23)**

#1 ‘circadian rhythm’/exp

#2 evening*

#3 morning*

#4 daytime

#5 afternoon

#6 night

#7 nighttime

#8 "time of day"

#9 immuniz*

#10 vaccines/ exp

#11 vaccination/ exp

#12 vaccin*

#13 #9 OR #10 OR #11 OR #12

#14 animal/exp

#15 human/exp

#16 #14 NOT #15

#17 #1 OR #2 OR #3 OR #4 OR #5 OR #6 OR #7 OR #8

#18 #17 AND #13

#19 #18 NOT #16

**Supplementary Information 2 - Tables**

Table S1 Studies excluded following full text screening

| Study ID | Reference | Reason for Exclusion |
| --- | --- | --- |
| Fleischer | Circadian variation of antibody formation after hepatitis B vaccination | Article in German |
| Du Pan | Can one use the circadian rhythm to minimize the febrile post vaccinal reactions of the baby | Reference cannot be sourced |
| Pollmann | Circadian variations of the efficiency of hepatitis B vaccination | Not possible to source original reference |
| Prather | Does sleep predict antibody response and maintenance to the covid-19 vaccine? | Abstract, no details of results or methods |
| Prather | Sleep and Antibody Response to Hepatitis B Vaccination | Time of vaccination not investigated |
| Presby | Biometrics from a wearable device reveal temporary effects of COVID-19 vaccines on cardiovascular, respiratory, and sleep physiology | Time of vaccination unclear, multiple vaccinations not specified. |
| Franck | Infant Sleep After Immunization: Randomized Controlled Trial of Prophylactic Acetaminophen | Time of vaccination unclear, multiple vaccinations not specified. |
| Zacks | Assessment of Predictors for SARS-CoV-2 Antibodies Decline Rate in Health Care Workers after BNT162b2 Vaccination—Results from a Serological Survey | Time of vaccination not investigated |
| Abbaspour | The effect of time of day of covid-19 vaccination and other covariates on side effects | Abstract, no details of results or methods |
| Mason | Metrics from Wearable Devices as Candidate Predictors of Antibody Response Following Vaccination against COVID-19: Data from the Second TemPredict Study | Time of vaccination not investigated |
| Föhse | The impact of circadian rhythm on Bacillus Calmette-Guérin vaccination effects on SARSCoV-2 infections | The effect of TODV cannot be interpreted because vaccine did not protect against SARSCoV-2 at either time point. |
| Dobaño | Eleven-month longitudinal study of antibodies in SARS-CoV-2 exposed and naïve primary health care workers upon COVID-19 vaccination. | No details on timing of vaccination |

Table S2 Methodology of included studies

| Study ID | Allocation to intervention (TODV) | Baseline immune status | Quantitative Outcome data | Summary of study conclusions |
| --- | --- | --- | --- | --- |
| Hazan | NI | No documented SARS-CoV-2 positive test prior to vaccination | Cox regression comparison of breakthrough infection after morning and afternoon vaccination compared to evening  Morning 0.95 (0.94-0.96) p < 0.001 Afternoon 0.92 (0.91-0.93) p < 0.001 Evening ref | Vaccination timing altered breakthrough infection risk by 8.6-25%. Highest rates in evening vaccination, lowest early afternoon. Non-linear relationship between time of vaccination and breakthrough infection, period 9-15h. Benefits more in elderly and young. ED visits not related to time of vaccination |
| Phillips (i) | Random* | Adjusted for baseline antibodies, but no information on previous vaccination/infection | No main effect of TODV on antibody response for hepatitis A vaccination  Sex x TODV interaction F(1,70) = 6.74, p = .01, ƞ = .088: men | No overall time of day or sex effect on antibody titre, morning higher only in men. Women had better response to afternoon vaccination, but this was not statistically significant. |
| Phillips (ii) | Self-selected | Adjusted for baseline antibodies, but no information on previous vaccination/infection | No main effect of TODV on antibody response for any vaccine response  Significant sex by TODV interaction only for A/Panama  Sex x TODV interaction F(1,84) = 5.93, p = .02, ƞ = .066: men | No time of day or sex main effect on antibody titre, after stratification. There was a sex x TODV interaction; men, but not women, mounted a better antibody response to morning vaccination but only for one strain. |
| Long | Random | Adjusted for baseline antibodies, and previous vaccination | Graphical data only | One of three grouped influenza strain vaccines associated with higher antibody response after morning administration. No effect of sex |
| Matryba | NI | No previous positive PCR test, seronegative | Antibody titre after dose 1 and 2. Mean (sd)  For am vs pm vaccination (NS)  am 102.48 (58.32) BAU/mL × 1000  pm 99.98 (59.41) BAU/mL × 1000 | No difference in antibody titre between morning and evening-vaccinated participants after 1 or 2 doses of vaccine. No difference in reported adverse effects after morning or evening vaccination |
| Jolliffe | NI | Seronegative | Risk of post-vaccination seronegativity, hazard ratio (95% CI) NS  12-2pm 1.21 (0.91, 1.61) ref 2pm-5pm 1.01 (0.78. 1.31) 5pm- 0.84 (0.57, 1.26)  Fully adjusted percentage difference between antibody titres after morning vaccination (NS) 12-2pm -2.87 (-6.00, 0.36)  2-5pm 1.15 (-1.16, 3.99) 5pm - -2.39 (-6.20, 1.58) | Time of day not associated with risk of seronegativity. Time of day of vaccination not associated with antibody titre levels 8 weeks later |
| Liu | Random | Adjusted for baseline antibodies, no information on previous vaccination or infection. | Fold change (mean (95% CI)) in antibody titres after vaccination (NS)  am  A/H1N1 3.82 (3.11, 4.69)  A/H3N2 2.23 (1.94, 2.55)  B/Victoria 1.57 (1.38, 1.78)  pm  A/H1N1 3.06 (2.48, 3.77) A/H3N2 2.12 (1.87, 2.40) B/Victoria 1.42 (1.27, 1.60) | No overall effect of TODV on antibody response or ADE. In subgroup analysis, participants aged over 65-75 years and in women, morning vaccination was associated with significantly higher antibody titres. |
| Whittaker (i) | Random* | No previous vaccination. Adjusted for baseline antibodies | Graphical data only | No significant association between TODV and any serotype. |
| Whittaker (ii) | Self selected | No previous vaccination. Adjusted for baseline antibodies | Graphical data only | No significant association between TODV and any serotype. Morning vaccination yielded a stronger response in the control group only. |
| Nachtigall | NI | NI | Odds of ADE, afternoon vs. morning (ref)  1.04 (0.96–1.12) (NS)  Odds of post vaccination incapacity to work  afternoon vs. morning (ref)  1.08 (0.97–1.19) (NS) | No association between TODV and impairment or time off work |
| Filippatos | NI | SARS-CoV-2 nucleocapsid negative | Antibody titre 1 month after 2nd dose (p = 0.006) median (IQR) 7am-11am 1,216 (1,193) U/ml 11am-3pm 1,287 (1,559) U/ml 3pm–10pm 1,744 (1,152) U/ml  Antibody titre 1 month after 3rd dose (p = 0.048) median (IQR) 7am-11am 17,885 (13,769) U/ml 11am-3pm 13,585 (13,267] U/ml 3pm-10pm 20,387 (14,155] U/ml  Antibody titre 1st, 4th and 8th months after vaccination NS  Changes in neutralising antibody titre dose not associated with TODV | Vaccination at 3pm-10pm associated with higher total antibodies after 1 month compared to other timepoints. Vaccination at 11am-3pm associated with lower total antibodies compared to other timepoints. No difference in tAbs-RBD after the first dose but only 1 month after the second and third dose. No association between neutralising antibodies and time of vaccination. No age interaction |
| Wang | NI | Seronegative for anti-n-SARS CoV2 at baseline, no positive PCR | Graphical data only | Antibody response higher in those vaccinated later in the day. No association between time of day of vaccination and non-responders. No interaction with sex or age or vaccine type. Distribution of TODV and blood sample collection was not consistent. |
| Langlois (i) | NI | Adjusted for baseline antibodies but no information on vaccination/infection history | Graphical data only | Antibody response to first dose of one of four strains tested varied by time of day (A/Philippines). No effect on revaccination. Local adverse reaction (arm soreness) more likely after mid-afternoon vaccination (14:30) |
| Langlois (ii) | NI | Adjusted for baseline antibodies but no information on vaccination/infection history | Graphical data only | No difference for any antigen related to time of day. Local adverse effects highest after 3pm vaccination only in revaccinated group. |
| Yamanaka | Self-selected | Seronegative SARS-CoV-2 nucleocapsid antigen | Post-vaccination antibody titre (Log10 BAU/mL) mean ± sd (NS)  13-20 days  am 2.40 ± 0.32  pm 2.44 ± 0.40  21-27 days  am 2.47 ± 0.31  pm 2.48 ± 0.31  28-45 days  am 2.33 ± 0.46  pm 2.47 ± 0.39  am 2.36 (LS-mean)  pm 2.42 (LS-mean)  Difference [95% CI]: 0.064 [2.41, 2.56] | No association between time of day of vaccination and mRNA 1273 vaccine antibody response |
| Gottlob | Random | Adjusted for baseline antibodies | *Haemophilus influenzae* IgG IU/ml, mean (sd)  am vaccination  Pre vaccination 0.3 (0.2)  Post-vaccination 0.7 (0.7)   pm vaccination Pre vaccination 0.3 (0.3)  Post-vaccination 0.9 (1.0) p < 0.05 | No difference in number of episodes related to time of day of vaccination. Increase in IgG to *Haemophilus influenzae* type V higher in evening compared to morning group |
| Abbaspour | NI | NI | Graphical data only | 6-11am and 4pm-10pm associated with more non-allergic adverse effects |
| Kurupati | NI | Adjusted for baseline antibodies and B-cell subsets but no information on vaccination history. | Graphical data only | Antibody titres higher after afternoon vaccination for one strain and in aged participants only, but this was explained by baseline differences |
| Karabay | Random | Seronegative | Anti-HBs (Geometric Mean Titre) (NS) am 923.3  pm 921.2 | No difference in antibody titre between morning and evening-vaccinated participants one month after 3rd booster |
| Erber | Administrator assignment | Seronegative, no previous vaccination | Risk of seronegativity 3 weeks post-vaccination. Adjusted OR (95% CI)* (BAU/mL) mean (sd) 09:00-10:00 Ref  10:00-11:00 7.8 (1.0, 64.2)  11:00-12:00 8.1 (1.0, 65.3)  12:00-13:00 16.4 (2.2, 123.4)  13:00-14:00 12.4 (1.6, 96.5)  14:00-15:00 4.8 (0.5, 47.5)  15:00-16:00 9.8 (1.1, 91.3)  *adjusted for age (continuous), sex and baseline antibody levels (continuous)  Antibody titre 3 weeks after vaccination (BAU/mL) mean (sd)  09:00-10:00 292.1 (262.1)  10:00-11:00 252.1 (253.4)  11:00-12:00 274.4 (259.4)  12:00-13:00 217.3 (226.4)  13:00-14:00 228.6 (236.9)  14:00-15:00 280.7 (266.2)  15:00-16:00 264.4 (262.4) | Non-linear relationship between time of vaccination and IgG levels. Morning and afternoon levels higher than mid-day. No interaction with age and sex. IgG levels lowest and odds of remaining seronegative highest between 12-2pm TODV. |
| de Bree | Evening and morning groups recruited separately | No previous vaccination. Adjusted for baseline data | Aggregate data not shown | Increased cytokine response to *S. aureus* in morning samples at 2 weeks or 3 months IL-1β and TNF-α different between baseline and 3 months in morning but not evening, IL-6 not different in either. IFNγ increased in morning group only at 3 months, and not in evening groups. More accessible chromatin in mTOR pathway genes in morning vaccination. |
| Zhang | Administrator assignment | Infection status before vaccination not ascertained. Adjusted for baseline antibodies | Neutralising Ab titre (AU/mL) median (IQR) am 34.70 (21.61, 43.59) pm 19.35 (11.83, 25.09)  Difference (95% CI) 14.84 (7.37, 24.15) | Morning vaccination higher nAb and stronger Tfh and B-cell response. Higher antibody secreting cells and CD138+ cells. Higher percentage monocytes and dendritic cells |
| Bohn-Goldbaum | NI | NI | Reaction types by TODV   Morning Afternoon  all 5.83 5.52  systemic 2.79 2.49  local 2.50 2.44 NS | Vaccine time of day not associated with reactivity |
| Feigin | NI | NI | Graphical data only | Diurnal patterns of blood amino acid disturbed in both morning and evening vaccine groups. Development of clinical signs of disease not different between TODV. Later and more persistent specific fluorescence in leukocytes peaked at 2 days in morning vaccination, and at 6-7 days in evening vaccination. |
| Pollman | NI | Seronegative | Not presented, graphical data | Afternoon vaccination induced higher antibody levels compared to morning. Local pain and swelling higher after afternoon vaccination. |
| Lai | Random | Negative PCR test within 1 week. Adjusted for baseline antibodies | Ab titre at 28 days post-vaccination compared to baseline, mean (sd) AU/ml (NS) am 22.2 (13.2, 45.0) pm 22.0 (14.4, 40.7) | No association between TODV and Ab titre or adverse events. No sex or age interactions |

NI No information

NS Not significant

* although participants were randomly allocated to morning or afternoon vaccination in these studies, 30% of them subsequently changed their appointment time, which invalidates the randomisation

procedure.

Table S3 Details of timing of vaccinations relative to sample collection and follow up.

| Study ID | Time of Day (am vaccination) | | | Time of Day (pm vaccination) | | | Circadian Alignment of Baseline and Follow-Up | Follow-up interval (days) |
| --- | --- | --- | --- | --- | --- | --- | --- | --- |
|  | *Pre-vaccine sample* | *Vaccination* | *Post-vaccine sample* | *Pre-vaccine sample* | *Vaccination* | *Post-vaccine sample* |  |  |
| Hazan | NA | 8am - 12pm | NA | NA | 12-4pm | NA | NA | 7-365 |
| Phillips (i) | 10am-12pm | 10am-12pm | NI | 4-6pm | 4-6pm | NI | NI | 31.3 (3.1) mean (sd) |
| Phillips (ii) | 8-11am | 8-11am | NI | 1-4pm | 1-4pm | NI | NI | 28.6 (2.91) mean (sd) |
| Long | 9-11am | 9-11am | Morning | 3-5pm | 3-5pm | Morning | No | 1 month |
| Matryba | NA | < 11am | 1-4pm | NA | > 3pm | 1-4pm | NA | 124.4 (8.7) mean (sd) |
| Jolliffe | NI | < 12pm | NI | NI | 12 - <5pm | NI | NI | 60.2 (44.8-70) median (IQR) |
| Liu | 8-10am | 9-11am | 8-10am | 8-10am | 3-5pm | 8-10am | Yes | 1 month, 3 months |
| Whittaker (i) | 10am-12pm | 10am-12pm | NI | NI | 4-6pm | NI | NI | 5 days (0.72 days), 28, 126 |
| Whittaker (ii) | Morning | Morning | NI | NI | Afternoon | NI | NI | 31 (4) and 183 (5) mean (sd) |
| Nachtigall | NA | Morning | NA | NA | Afternoon | NA | NA | NI |
| Filippatos | NA | 7-11am | 8-4pm | NA | 11am-3pm | 8-4pm | No | 21 (dose 1) 1, 4, 8 (dose 2), 1 (dose 3) months |
| Wang | NI | 7-11am | 9am-9pm | NI | 11am-3pm | 9am-9pm | No | 14-70 |
| Langlois (i) | Continuous | Continuous | NI | Continuous | Continuous | NI | NI | 21-28 |
| Langlois (ii) | Continuous | Continuous | NI | Continuous | Continuous | NI | NI | 21-28 |
| Yamanaka | NI | Morning | NI | NI | Afternoon | NI | NI | 13-20, 21-27 and 28-45 |
| Gottlob | NI | 7-10am | 7-10am | NI | 7pm -10pm | 7pm -10pm | NI | NI |
| Abbaspour | NA | 6-11am | NA | NA | 11am-4pm | NA | NA | 1,2 3 |
| Kurupati | 8-12pm | 8-12pm | Self selected | 12-5pm | 12pm-5pm | Self selected | No | 7, 14 and 28 |
| Karabay | NI | 8-8:30am | NI | NI | 5:30pm-6pm | NI | NI | 1 month |
| Erber | Continuous | Continuous | 9am-12pm | Continuous | Continuous | 9am-12pm | No | 21 |
| de Bree | 8-12pm | 8am-12pm | 8-12pm | 8am-12pm | 6pm-6.30pm | 8-12pm | Yes | 28, 3 months |
| Zhang | 8-10am | 9-11am | 8-10am | 3-5pm | 3-5pm | 3-5pm | Yes | 14, 21, 28, 56 |
| Bohn-Goldbaum | NA | Morning | NA | NA | Evening | NA | NA | 3 days and fortnights 1-8 |
| Feigin | NA | 8am | 8am and 8pm | NA | 8pm | 8am and 8pm | Yes | 1-11 |
| Pollman | NA | 7:30-9am | NI | NA | 1-3pm | NI | NI | 4-8 months |
| Lai | 8-10am | 9-11am | 8-10am | 8-10am | 3-5pm | 8-10am | Yes | 28 |

NI No information

**References**

Abbaspour S, Wang W, Robbins G and Klerman E (2022) The effect of time of day of Covid-19 vaccination and other covariates on side effects. Sleep 45:A95–A96.

Abbaspour S, Robbins GK, Blumenthal KG, Hashimoto D, Hopcia K, Mukerji SS, Shenoy ES, Wang W, and Klerman EB (2022) Identifying Modifiable Predictors of COVID-19 Vaccine Side Effects: A Machine Learning Approach. Vaccines 10:1747.

Bohn-Goldbaum E, Cross T, Leeb A, Peters I, Booy R and Edwards KM (2022) Adverse events following influenza immunization: Understanding the role of age and sex interactions. Expert Rev Vaccines 21:415–422.

de Bree LCJ, Mourits VP, Koeken VACM, Moorlag SJCFM, Janssen R, Folkman L, Barreca D, Krausgruber T, Fife-Gernedl V, Novakovic B, Arts RJW, Dijkstra H, Lemmers H, Bock C, Joosten LAB, van Crevel R, Benn CS and Netea MG (2020) Circadian rhythm influences induction of trained immunity by BCG vaccination. J Clin Invest 130:5603–5617.

Dobaño C, Ramírez-Morros A, Alonso S, Ruiz-Olalla G, Rubio R, Vidal M, Prados de la Torre E, Jairoce C, Mitchell RA, Barrios D, Jiménez A, Rodrigo Melero N, Carolis C, Izquierdo L, Zanoncello J, Aguilar R, Vidal-Alaball J, Moncunill G and Ruiz-Comellas A (2022) Eleven-month longitudinal study of antibodies in SARS-CoV-2 exposed and naïve primary health care workers upon COVID-19 vaccination. Immunology 167:528–543.

Erber AC, Wagner A, Karachaliou M, Jeleff M, Kalafatis P, Kogevinas M, Pepłońska B, Santonja I, Schernhammer E, Stockinger H, Straif K, Wiedermann U, Waldhör T and Papantoniou K (2023) The Association of Time of Day of ChAdOx1 nCoV-19 Vaccine Administration With SARS-CoV-2 Anti-Spike IgG Antibody Levels: An Exploratory Observational Study. J Biol Rhythms 38:98–108.

Feigin RD (1967) Live attenuated Venezuelan equine encephalomyelitis virus vaccine. Am J Trop Med Hyg 16:1476-1645.

Filippatos F, Tatsi E-B, Efthymiou V, Syriopoulou V and Michos A (2022) Time of Day of BNT162b2 COVID-19 Immunization Affects Total SARS-CoV-2 Antibody Levels but Not Neutralizing Activity. J Biol Rhythms 37:562–566.

Fleischer B (1946) Circadian variation of antibody formation after hepatitis B vaccination. Deutsche medizinische Wochenschrift 118:999

Föhse K, Taks EJM, Moorlag SJCFM, Bonten MJM, van Crevel R, ten Oever J, van Werkhoven CH, Netea MG, van de Maat JS and Hoogerwerf JJ (2023) The impact of circadian rhythm on Bacillus Calmette-Guérin vaccination effects on SARS-CoV-2 infections. Front Immunol 14:980711.

Franck L, Gay CL, Lynch M and Lee KA (2011) Infant Sleep After Immunization: Randomized Controlled Trial of Prophylactic Acetaminophen. Pediatrics 128:1100–1108.

Gottlob S, Gille C and Poets CF (2019) Randomized Controlled Trial on the Effects of Morning versus Evening Primary Vaccination on Episodes of Hypoxemia and Bradycardia in Very Preterm Infants. Neonatology 116:315–320.

Hazan G, Duek OA, Alapi H, Mok H, Ganninger AT, Ostendorf EM, Gierasch C, Chodick G, Greenberg D and Haspel JA (2023) Biological rhythms in COVID-19 vaccine effectiveness in an observational cohort study of 1.5 million patients. J Clin Invest 133:e167339.

Jolliffe DA, Faustini SE, Holt H, Perdek N, Maltby S, Talaei M, Greenig M, Vivaldi G, Tydeman F, Symons J, Davies GA, Lyons RA, Griffiths CJ, Kee F, Sheikh A, Shaheen SO, Richter AG and Martineau AR (2022) Determinants of Antibody Responses to SARS-CoV-2 Vaccines: Population-Based Longitudinal Study (COVIDENCE UK). Vaccines 10:1601.

Karabay O, Temel A, Koker AG, Tokel M, Ceyhan M and Kocoglu E. (2008) Influence of circadian rhythm on the efficacy of the hepatitis B vaccination. Vaccine 26:1143–1144.

Kurupati RK, Kossenkoff A, Kannan S, Haut LH, Doyle S, Yin X, Schmader KE, Liu Q, Showe L and Ertl HCJ (2017) The effect of timing of influenza vaccination and sample collection on antibody titers and responses in the aged. Vaccine 35:3700–3708.

Lai F, Li B, Mei J, Zhou Q, Long J, Liang R, Mo R, Peng S, Liu Y and Xiao H (2023) The Impact of Vaccination Time on the Antibody Response to an Inactivated Vaccine against SARS‐CoV‐2 (IMPROVE‐2): A Randomized Controlled Trial. Adv Biol 2300028. 34.

Whittaker AC, Gallagher S and Drayson M (2022) Time of day of vaccination does not relate to antibody response to thymus-independent vaccinations. Vaccine: X 11, 100178.

Langlois PH, Smolensky MH, Glezen WP and Keitel WA (1995) Diurnal Variation in Responses to Influenza Vaccine. Chronobiol Int 12:28–36.

Liu Y, Zhang H, Yuan G, Yao M, Li B, Chen J, Fan Y, Mo R, Lai F, Chen X, Li M, Chen B, Lord JM, Peng S, Cheng K and Xiao H (2022) The impact of circadian rhythms on the immune response to influenza vaccination in middle-aged and older adults (IMPROVE): A randomised controlled trial. Immun Ageing 19:46.

Long JE, Drayson MT, Taylor AE, Toellner KM, Lord JM and Phillips AC (2016) Morning vaccination enhances antibody response over afternoon vaccination: A cluster-randomised trial. Vaccine 34:2679–2685.

Mason AE, Kasl P, Hartogensis W, Natale JL, Dilchert S, Dasgupta S, Purawat S, Chowdhary A, Anglo C, Veasna D, Pandya LS, Fox LM, Puldon KY, Prather JG, Gupta A, Altintas I, Smarr BL, Hecht FM (2022) Metrics from Wearable Devices as Candidate Predictors of Antibody Response Following Vaccination against COVID-19: Data from the Second TemPredict Study. Vaccines 10:264

Matryba P, Gawalski K, Ciesielska I, Horvath A, Bartoszewicz Z, Sienko J, Ambroziak U, Malesa-Tarasiuk K, Staniszewska A, Golab J and Krenke R (2022) The Influence of Time of Day of Vaccination with BNT162b2 on the Adverse Drug Reactions and Efficacy of Humoral Response against SARS-CoV-2 in an Observational Study of Young Adults. Vaccines 10:443.

Nachtigall I, Bonsignore M, Hohenstein S, Bollmann A, Günther R, Kodde C, Englisch M, Ahmad-Nejad P, Schröder A, Glenz C, Kuhlen R, Thürmann P and Meier-Hellmann A (2022) Effect of gender, age and vaccine on reactogenicity and incapacity to work after COVID-19 vaccination: A survey among health care workers. BMC Infectious Diseases 22:291.

Phillips AC, Gallagher S, Carroll D and Drayson M (2008) Preliminary evidence that morning vaccination is associated with an enhanced antibody response in men. Psychophysiology 45:663–666.

Pollmann L and Pollmann B (1988) Circadian variations of the efficiency of hepatitis B vaccination. Ann Rev Chronopharmacol 5:45–48.

Prather A, Epel E, Drury S and Robinson J (2022) Does sleep predict antibody response and maintenance to the COVID-19 vaccine? Sleep 45:A92

Prather AA, Hall M, Fury JM, Ross DC, Muldoon MF, Cohen S, Marsland AL (2012) Sleep and Antibody Response to Hepatitis B Vaccination. Sleep 35:1063-9.

Presby DM and Capodilupo ER (2022) Biometrics from a wearable device reveal temporary effects of COVID-19 vaccines on cardiovascular, respiratory, and sleep physiology. J Appl Physiol 132:448–458.

Wang W, Balfe P, Eyre DW, Lumley SF, O’Donnell D, Warren F, Crook DW, Jeffery K, Matthews PC, Klerman EB and McKeating JA (2022) Time of Day of Vaccination Affects SARS-CoV-2 Antibody Responses in an Observational Study of Health Care Workers. J Biol Rhythms 37:124-129.

Whittaker AC, Gallagher S and Drayson M (2022) Time of day of vaccination does not relate to antibody response to thymus-independent vaccinations. Vaccine: X 11:100178. Yamanaka Y, Yokota I, Yasumoto A, Morishita E and Horiuchi H (2022) Time of Day of Vaccination Does Not Associate With SARS-CoV-2 Antibody Titer Following First Dose of mRNA COVID-19 Vaccine. J Biol Rhythms 37:700–706.

Zacks N, Shai A, Levi H, Breslavsky A, Maayan S, Evgenia T, Feitelovich S, Wand S, Schaffer M, Sherer Y, Givaty G, Ferber , Tal M, Bilenko N (2022) Assessment of Predictors for SARS-CoV-2 Antibodies Decline Rate in Health Care Workers after BNT162b2 Vaccination—Results from a Serological Survey. Vaccines 10:1443.

Zhang H, Liu Y, Liu D, Zeng Q, Li L, Zhou Q, Li M, Mei J, Yang N, Mo S, Liu Q, Liu M, Peng S and Xiao H (2021) Time of day influences immune response to an inactivated vaccine against SARS-CoV-2. Cell Res 31:1215–1217.
